# Epidemiological profiles and associated risk factors of SARS-CoV-2 positive patients based on a high-throughput testing facility in India

**DOI:** 10.1098/rsob.200288

**Published:** 2021-06-02

**Authors:** Sumit Malhotra, Manju Rahi, Payal Das, Rini Chaturvedi, Jyoti Chhibber-Goel, Anup Anvikar, Hari Shankar, C. P. Yadav, Jaipal Meena, Shalini Tewari, Sudha V. Gopinath, Reba Chhabra, Amit Sharma

**Affiliations:** ^1^ Centre for Community Medicine, All India Institute of Medical Sciences, New Delhi 110029, India; ^2^ Division of Epidemiology and Communicable Diseases, Indian Council of Medical Research, New Delhi 110029, India; ^3^ Molecular Medicine, International Centre for Genetic Engineering and Biotechnology, New Delhi 110067, India; ^4^ ICMR-National Institute of Malaria Research, New Delhi 110077, India; ^5^ National Institute of Biologicals, Institutional Area, Noida, Uttar Pradesh 201309, India

**Keywords:** COVID-19, clinical profile, India, risk factors

## Abstract

We describe the epidemiological characteristics and associated risk factors of those presenting at a large testing centre for SARS-CoV-2 infection. This is a retrospective record review of individuals who underwent SARS-CoV-2 testing by reverse transcription–polymerase chain reaction (RT-PCR) at a high-throughput national-level government facility located in the north of India. Samples collected from 6 April to 31 December 2020 are included in this work and represent four highly populous regions. Additionally, there was a prospective follow-up of 1729 cases through telephone interviews from 25 May 2020 to 20 June 2020. Descriptive analysis has been performed for profiling clinic-epidemiological aspects of suspect cases. Multivariable logistic regression analysis was undertaken to determine risk factors that are associated with SARS-CoV-2 test positivity and symptom status. A total of 125 600 participants' details have been included in this report. The mean (s.d.) age of the participants was 33.1 (±15.3) years and 66% were male. Among these tested, 9515 (7.6%) were positive for COVID-19. A large proportion of positive cases were asymptomatic. In symptomatic positive cases, the commonest symptoms were cough and fever. Increasing age (groups 20–59 and ≥60 years compared to age group less than 5 years), male sex, history of international travel, symptoms for SARS-CoV-2, and participants from Delhi and Madhya Pradesh were positively associated with SARS-CoV-2 test positivity. Having co-morbidity, risk behaviours and intra-familial positivity were associated with a positive odds ratio for exhibiting SARS-CoV-2 symptoms. Intensified testing and isolation of cases, identification of both asymptomatic and symptomatic individuals and additional care of those with co-morbidities and risk behaviours will all be collectively important for disease containment in India. Reasons for differentials in testing between men and women remain an important area for in-depth study. The increased deployment of vaccines is likely to impact the trajectory of COVID-19 in the coming time, and therefore our data will serve as a comparative resource as India experiences the second wave of infection in light of newer variants that are likely to accelerate disease spread.

## Graphical abstract

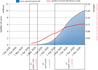

## 1. Introduction

The first case of novel coronavirus disease in India was reported in a student from Thrissur, Kerala, who returned from Wuhan, China, on 30 January 2020 [1]. Later on, cases were reported from other parts of the country which were mostly either connected with the recent history of international travel or with an exposure to a confirmed case of COVID-19. India had reported the largest number of confirmed COVID-19 cases in Asia and ranked second worldwide after the United States during the first wave. As of 30 April 2021, India has confirmed a total of greater than 16.9 million cases with 192 311 deaths attributed to COVID-19 [2]. India is now dealing with an explosive second wave of SARS-CoV-2 infections and has the dubious distinction of largest daily cases worldwide. The brutal second wave of COVID-19 has hit the nation reporting more than 0.3 million cases daily since mid-April 2021. Possible reasons for the second wave in India include: large susceptible population, presence of virulent mutant strains of the virus, very poor planning and roll-out of vaccination for its masses, indications that India was achieving herd immunity led to complacency in measures, congregation of masses in religious gatherings and political rallies, pandemic fatigue and lowering of guard due to the false propaganda that COVID had been defeated. The rise of the second wave is much steeper than the first wave that peaked in September of 2020. Although this study is restricted to the fast wave of the Indian epidemic, our analyses will enlighten data from the second and future waves of SARS-CoV-2 infections.

At the onset of the pandemic, the government of India carried out testing of the exposed people for SARS-CoV-2 based on set criteria which underwent periodic changes in light of the evolving scenario of the pandemic. The criteria for testing were first laid down on 17 March and a nationwide lockdown was implemented on 24 March 2020. The laboratory testing capacity for SARS-CoV-2 has been ramped up since the initial lockdown in the country and it stands at 2501 laboratories as of 30 April 2021 [3] with a testing rate of 1418 daily tests per million population. India had nearly 253 daily confirmed cases per million and its daily positive rate was 21% as of 30 April 2021 [4].

Internationally, there is limited information from SARS-CoV-2 testing centres in regard to the socio-demographic profiles of those who got tested. Countries like Brazil, China, Italy, the UK and the USA have mapped the clinical and epidemiological features of patients with COVID-19. Docherty *et al.* [5] performed a large prospective cohort study and characterized the clinical features of 20 133 patients who were admitted to hospital with COVID-19 in the UK. The median age of patients admitted to hospital with COVID-19 or diagnosed in hospital was 73 years. More men were admitted than women. The commonest co-morbidities were chronic cardiac disease, uncomplicated diabetes, non-asthmatic chronic pulmonary disease and chronic kidney disease. Within an Asian setting, Huang *et al.* [6] have reported the epidemiological, clinical, laboratory, radiological and clinical features of patients in Wuhan, China. Most of the infected patients were men; less than half had underlying diseases including diabetes, hypertension and cardiovascular disease with a median age was 49 years. Common symptoms at the onset of illness were fever, cough and myalgia or fatigue. Grasseli *et al.* [7] have characterized the patients with COVID-19 symptoms in the Lombardy region of Italy. Of the 1591 patients included in the study, the median age was 63 years and 1304 (82%) were males. Of the 1043 patients with available data, 709 (68%) had at least one co-morbidity and 509 (49%) had hypertension.

We report here clinic-epidemiological features along with the risk factors among the positive patients in one of the largest cohort of potential COVID-19 cases (*n* = 125 600) who were tested through reverse transcription-polymerase chain reaction (RT-PCR) for the detection of SARS-CoV-2 infection from the period of 6 April 2020 to 31 December 2020 at National Institute of Biologicals (NIB), an autonomous institute of Ministry of Health and Family Welfare situated in Noida, Uttar Pradesh, India (figure 2). The deployment of vaccines is likely to impact the trajectory of COVID-19 in the coming time, and therefore our data will serve as a comparative resource as the pandemic continues, especially in light of newer variants or mutant strains that can accelerate disease spread.

## 2. Material and methods

### 2.1. Study design and data collection

**Figure 1.**
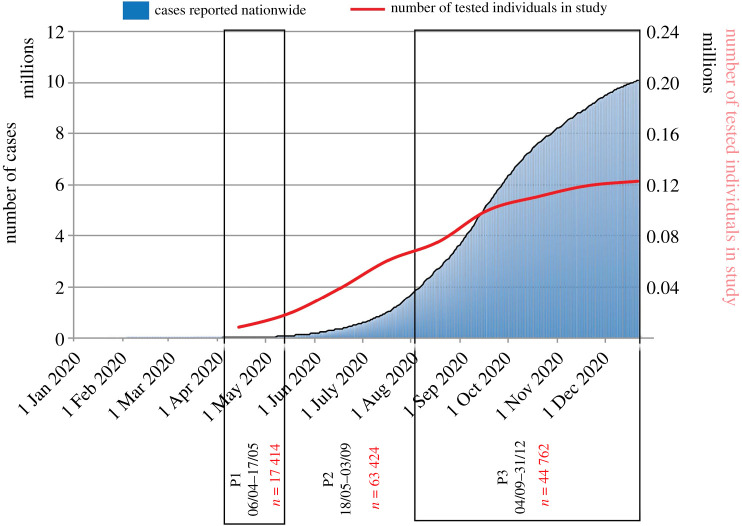
Cumulative COVID-19 positive cases since the first case on 30 January 2020 nationwide. The portion of the histogram shows the policy change dates for COVID-19 (P1: 06/04–17/05; P2: 18/05–03/09; P3: 04/09–31/12). The blue line on the graph represents daily positive cases (moving average). Data for COVID-19 were adapted from the World Health Organization Coronavirus Disease Dashboard. The red line on the graph represents the cumulative suspected number of cases from the database from the current study. Policy changes in different time periods were: P1—all symptomatic patients with international travel history, contacts with positive cases, and healthcare workers managing COVID-19 positive patients to be tested; P2—in addition to P1, all healthcare/frontline workers involved in containment and mitigation of COVID-19, all hospitalized patients who developed ILI symptoms, and all symptomatic among returnees and migrants within 7 days of illness to be tested; P3—routine surveillance in containment by rapid diagnostic tests, exposed and asymptomatic, symptomatic patients and high-risk patients (those requiring hospitalization with co-morbidities/elderlies ≥ 65 years/immunocompromised/pregnant females) to be tested by molecular techniques, and it also allowed self-referrals.

**Figure 2.**
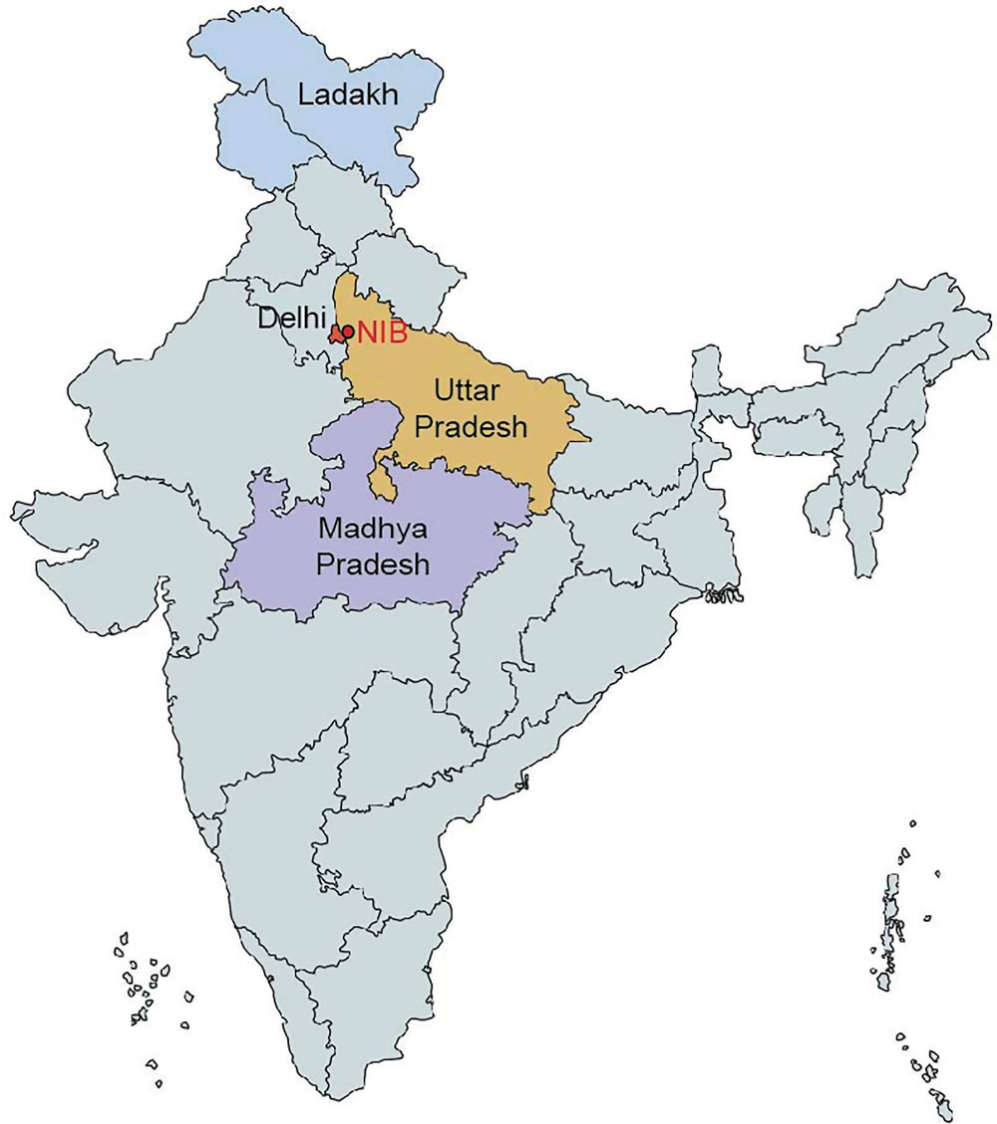
Map showing the location of National Institute of Biologicals (NIB), Noida, and four states from where samples were received.

The study has two components. The first is a retrospective analysis of the data from individuals who were tested for SARS-CoV-2 at NIB within north India. A total of 130 132 samples were tested in the period from 6 April 2020 to 31 December 2020. Considering missing information about demographic variables, symptom status, test results and repeat samples, a total of 125 600 individuals were included in this study. In addition, a subset of positive cases was followed up prospectively by telephonic interviews to enquire about the symptomatic status, morbidity profile and outcome. The required information was collated from 1729 positive cases from the period from 25 May 2020 to 20 June 2020. The individuals included in this study were people who were suspected to be exposed to a confirmed case of COVID-19, symptomatic frontline workers, symptomatic who had undertaken international travel, and those presented for laboratory testing from containment zones, or quarantine centres or self-isolation. The government of India brought in the first guidelines for testing on 17 March 2020 which mandated that the following individuals be tested: all symptomatic individuals (having cough, fever, difficulty in breathing) who (a) had undertaken international travel in the past 14 days or (b) had contacts of laboratory-confirmed cases, and (c) health workers managing COVID-19 patients. Shortly after the first guidelines (on 20 March and 9 April 2020), the testing criteria were broadened to include patients with severe acute respiratory illnesses (SARI) and with influenza-like illness (ILI) belonging to hotspots and gatherings. Major changes in the testing strategy were brought in during the months of May and September 2020 [8,9]. The guidelines released on 18 May 2020 in addition to the existing guidance identified all symptomatic individuals with SARI and ILI, contacts and migrants as eligible for testing. SARI and ILI were defined clinically. Another major testing strategy change was introduced on 4 September 2020 in which routine surveillance in containment by rapid antigen test was introduced [9]. In this policy, all patients of ILI/SARI and asymptomatic high-risk patients in a hospital or requiring hospital due to any co-morbidities (age ≥65 years/immunocompromised status/pregnant women in or near labour, etc.) had to be tested by RT-PCR/TrueNat/CBNAAT. In addition, the guidelines permitted people to self-refer and get themselves tested for SARS-CoV-2 without any prescription. This allowed for on-demand and wider testing. In view of the above time points, we have referred to our data in different periods for ease of analysis as P1 (from 6 April to 17 May 2020), P2 (from 18 May to 3 September 2020) and P3 (4 September to 31 December 2020) (figure 1). Majorly, the individuals who belonged to three neighbouring states of Delhi, Uttar Pradesh and Madhya Pradesh were identified as per government regulations for testing; additionally, there were 290 samples (0.2%) from Ladakh. During the initial time of this study, NIB was the only centre with high-throughput RT-PCR and hence these Indian states sent their samples to this laboratory for processing (figure 2). Epidemiological data pertaining to demographic characteristics, clinical presentation/symptoms, co-morbidities, hospitalization details, recent travel history, case referral state, etc. were recorded on the Specimen Referral Form (SRF) as per standards laid out by the Indian Council of Medical Research (ICMR), Ministry of Health and Family Welfare, Government of India [10].

**Figure 3.**
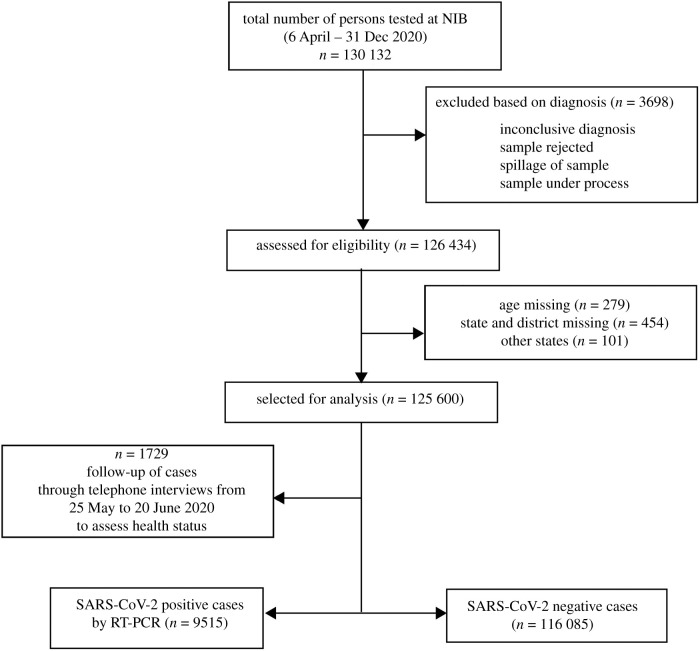
Study flow chart.

The second part of the study involved a prospective follow-up of individuals who were found to be SARS-CoV-2 positive during the period 6 April to 7 June 2020. We intended to follow-up all 2158 positive cases telephonically and were successful in 1729 cases (i.e. response rate of 80%). The missing 429 positive individuals could not be followed due to wrong/invalid numbers and/or unwillingness to respond. The telephone interviews for the 1729 cases were carried out from 25 May 2020 to 20 June 2020 to assess their health status (figure 3). A questionnaire was designed to collect the information related to family members and their COVID-19 status, lifestyle habits such as tobacco smoking and alcohol intake, course of the disease, hospitalization and recovery. Since the mode of the interview was telephonic, participants’ height and weight could not be measured directly to define their obesity status, but proxy indicators were collected in the form of self-perceived status about weight (normal, lean, overweight) and height (normal, small and tall stature).

### 2.2. Laboratory procedures

Trained personnel collected nasopharyngeal and oropharyngeal samples using standard guidelines laid out by the Indian Council of Medical Research (ICMR) [11]. Both types of swabbed samples were placed in a single viral transport medium (VTM) tube, packed in a triple-layered casing and transported under cold chain maintenance to the National Institute of Biologicals (NIB), Noida, Uttar Pradesh, India, that created a Biosafety level-2 (BSL-2) laboratory under negative pressure. A total of 600 microlitres of sample in VTM was transferred in barcoded secondary tubes which were fed to the system. A fully automated high-throughput M/s Roche Cobas 6800 system was used for COVID-19 testing based on the detection of the viral genome using real-time RT-PCR based diagnosis. Cobas SARS-CoV-2 diagnostic kits were used, which were a real-time RT-PCR two target test intended for the qualitative detection of nucleic acids from SARS-CoV-2 in nasopharyngeal/ oropharyngeal samples collected in VTM. Limit of detection studies determine the lowest detectable concentration of SARS-CoV-2 at which at least 95% of all (true positive) replicates test positive. The concentration level with observed hit rates greater than or equal to 95% were 0.009 and 0.003 TCID_50_/mL for SARS-CoV-2 (Target 1) and pan-Sarbecovirus (Target 2), respectively. Cobas processed the samples in batches of 96 including one positive and one negative control. The Cobas 6800 machine could test up to 940 samples in a day under standard operational conditions. The output of the tests was interpreted according to the chart described elsewhere [12].

### 2.3. Statistical analysis

The RT-PCR positive and negative data were entered in Microsoft Excel and line lists were prepared for all cases. Lists of quality checks were applied to ensure data quality. The dataset was locked and subjected to analysis using statistical software STATA Version 12.0 (StataCorp LP, College Station, TX 77845, USA) where in descriptive analyses were performed. Categorical variables were reported as frequency and proportions, and continuous variables were summarized as mean and standard deviation (s.d.). We also examined changes with respect to variables over three testing periods (P1, P2, P3). Multivariable logistic regression analysis was undertaken for two outcome variables: SARS-CoV-2 test positivity and clinical symptom positivity among the cases followed up. Symptom positivity was considered when SARS-CoV-2 was positive for any of the symptoms listed by ICMR [13]. A range of explanatory variables was included and their adjusted odds ratios along with 95% confidence intervals were calculated. The *p*-values < 0.05 were considered statistically significant.

## 3. Results

### 3.1. Profiles of participants

The study flow chart is shown in figure 3. A total of 125 600 individuals were tested at NIB whose details were retrieved from records and included in our study. The characteristics of participants included in the study are shown in table 1. The mean age in years (s.d.) of participants was 33.1 (±15.3) years. The dataset included all age groups with the majority being in the age band of 20–59 years (77%), followed by 6–19 years (14%) and ≥60 years (7%). Sex details were missing for six individuals.

**Table 1.** Participant characteristics across age categories.

characteristics	age category				total
	≤5 years	6–19 years	20–59 years	≥60 years	
*n* (%)	2848 (2.3)	17 379 (13.8)	96 947 (77.2)	8426 (6.7)	125 600 (100.0)
mean ± s.d. (years)	3.3 (±1.4)	14.1 (±4.0)	34.6 (±10.5)	66.2 (±6.7)	33.1 (±15.3)
gender					
females (%)	1223 (43.0)	6235 (35.9)	32 421 (33.4)	2900 (34.4)	42 779 (34.1)
males (%)	1624 (57.0)	11 144 (64.1)	64 521 (66.6)	5526 (65.6)	82 815 (65.9)
international travel					
no (%)	2841 (99.7)	17 350 (99.8)	96 616 (99.7)	8400 (99.7)	125 207 (99.7)
yes (%)	7 (0.3)	29 (0.2)	331 (0.3)	26 (0.3)	393 (0.3)
institutional quarantine					
no (%)	2256 (79.2)	13 892 (79.9)	79 522 (82.0)	6954 (82.5)	102 624 (81.7)
yes (%)	592 (20.8)	3487 (20.1)	17 425 (18.0)	1472 (17.5)	22 976 (18.3)
state of residence					
Uttar Pradesh (%)	2223 (78.1)	14 414 (82.9)	81 206 (83.8)	6901 (81.9)	104 744 (83.4)
Delhi (%)	528 (18.5)	2641 (15.2)	12 973 (13.4)	1203 (14.3)	17 345 (13.8)
Madhya Pradesh (%)	81 (2.8)	276 (1.6)	2556 (2.6)	308 (3.7)	3221 (2.6)
Ladakh (%)	16 (0.6)	48 (0.3)	212 (0.2)	14 (0.2)	290 (0.2)
co-morbidity					
no (%)	2847 (99.96)	17 372 (99.96)	96 696 (99.7)	8361 (99.2)	12 5276 (99.7)
yes (%)	1 (0.04)	7 (0.04)	251 (0.3)	65 (0.8)	324 (0.3)
symptomatic status					
asymptomatic	2762 (96.9)	16 958 (97.6)	93 617 (96.6)	8083 (95.9)	12 1420 (96.7)
symptomatic	86 (3.0)	421 (2.4)	3330 (3.4)	343 (4.1)	4180 (3.3)
hospitalization					
no (%)	2695 (97.8)	16 771 (99.4)	93 272 (99.4)	8036 (99.0)	120 774 (99.3)
yes (%)	62 (0.2)	102 (0.6)	587 (0.6)	78 (1.0)	829 (0.7)
RT-PCR SARS-CoV-2					
negative	2695 (94.6)	16 302 (93.8)	89 495 (92.3)	7593 (90.1)	116 085 (92.4)
positive	153 (5.4)	1077 (6.2)	7452 (7.7)	833 (9.9)	9515 (7.6)

Two-thirds of samples (66%) were collected from men and less than 1% had a recent history of international travel in past one/two months (table 1). Institutional quarantine as a measure of curtailing transmission was self-reported by 18% of individuals (table 1). The samples were received from the state of Uttar Pradesh (83%) followed by the national capital, Delhi (14%). The distribution of characteristics in tested individuals as per three periods of varying inclusion criteria of testing (referred as P1 to P3 in this paper, as explained earlier) is shown in table 2. No major change was seen in the percentage of different age categories that got tested across three policy periods except in the last period (P3) where the proportion of 6–19 years that were tested increased from 11.8% in P1 to 17.6% in P3. A slight increase in proportion among those tested in P3 was also seen for the elderly age group. We also observed an increase in the proportion of women being tested from periods P1 to P3. Self-report of institutional quarantine decreased over time frames of P1 to P3.

**Table 2.** Profile of participants over three testing periods.

	Period 1 (P1) 06/04/20 to 17/05/20 (*n* = 17 414)	Period 2 (P2) 18/05/20 to 03/09/20 (*n* = 63 424)	Period 3 (P3) 04/09/20 to 31/12/20 (*n* = 44 762)	*p*-value
age				
≤5 years	359 (2.1)	1315 (2.1)	1174 (2.6)	<0.001
6–19 years	1995 (11.5)	7501 (11.8)	7883 (17.6)	
20–59 years	13 873 (79.7)	50 580 (79.7)	32 494 (72.6)	
≥60 years	1187 (6.8)	4028 (6.3)	3211 (7.2)	
gender				
female	4440 (25.5)	20 378 (32.1)	17 961 (40.1)	<0.001
male	12 974 (74.5)	43 046 (67.9)	26 795 (59.9)	
institutional quarantine				
no	15 221 (87.4)	48 443 (9.6)	38 960 (5.1)	<0.001
yes	2193 (12.6)	14 981 (8.4)	5802 (2.9)	
state				
Delhi	6174 (35.4)	59 (0.1)	11 112 (24.8)	<0.001
Madhya Pradesh	3221 (18.5)	0	0	
Uttar Pradesh	8019 (46.0)	63 075 (99.4)	33 650 (75.2)	
Ladakh	0	290 (0.5)	0	
clinical symptoms				
no	15 466 (88.8)	62 860 (99.1)	43 094 (96.3)	<0.001
yes	1948 (11.2)	564 (0.9)	1668 (3.7)	
RT-PCR SARS-CoV-2				
no	15 994 (91.8)	57 510 (90.7)	42 581 (95.1)	<0.001
yes	1420 (8.1)	5914 (9.3)	2181 (4.9)	

### 3.2. Positivity rate and COVID-19 characteristics

**Figure 4.**
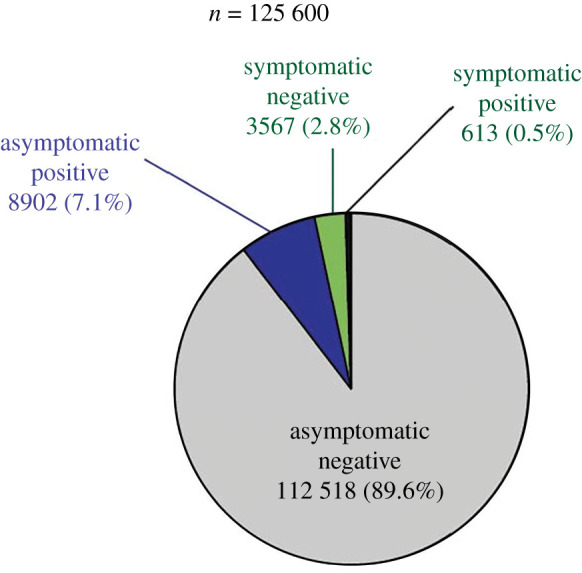
Distribution of a total number of individuals tested for SARS-CoV-2 (*n* = 125 600). The pie-chart shows the distribution of individuals tested, and the positives and negatives for SARS-CoV-2 along with symptom status.

**Figure 5.**
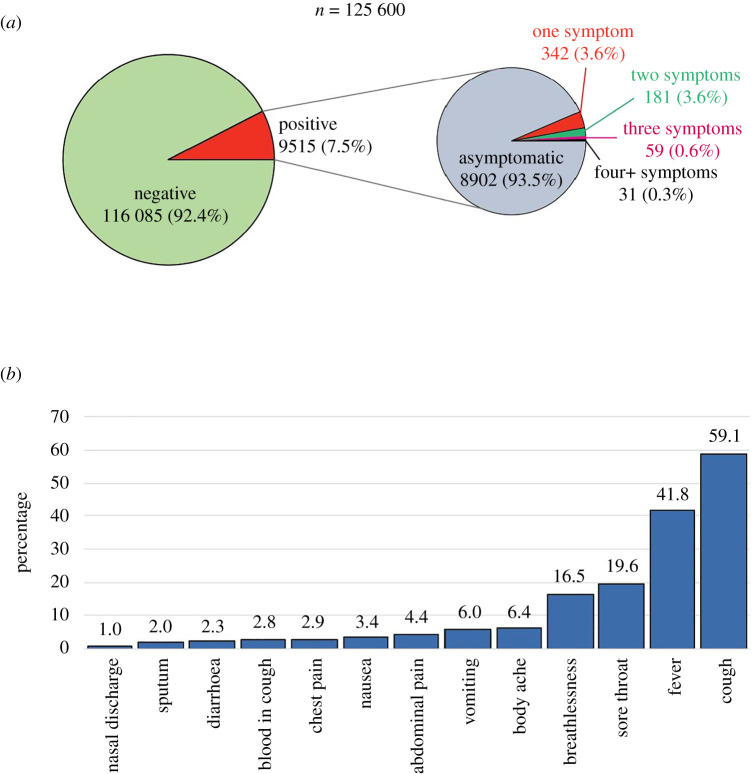
COVID-19 patients with respect to their symptoms. (*a*) A total number of patients who presented themselves with symptoms (*n* = 125 600) and (*b*) proportion with symptoms (*n* = 613). Note: The symptoms for *n* < 5 have been considered as others.

Of 125 600, a total of 9515 (7.6%) individuals were found to be positive for SARS-CoV-2 by RT-PCR. There was a slight increase in positivity from P1 (8%) to P2 (9%) followed by a decrease in positivity rate (5%) among our tested samples (table 2). The distribution of cases and their characteristics are shown in table 3. At the time of testing, 4180 (3.3%) reported having COVID-19-like symptoms. A total of 90% (112 518) of individuals were asymptomatic negative, 7% (8902) were asymptomatic positive, 3% (3567) were symptomatic negative and 0.5% (613) were symptomatic positive, as shown in figure 4. The percentage of symptomatic patients among suspect COVID-19 cases was higher in P1 (11%) and then a huge decrement was observed in P2 (1%) and finally a only small increase was observed in P3 (4%). Overall, among all 9515 COVID-19 positives, 94% were asymptomatic and only the remaining 6% had one or more symptoms. The distribution of all cases among age categories as ≤5, 6–19, 20–59 and ≥60 years was 2% (153), 11% (1077), 78% (7452) and 9% (833), respectively, where approximately 68% (6432) were men. With respect to their residences, 79% (7513), 17% (1578) and 4% (418) were from Uttar Pradesh, Delhi and Madhya Pradesh, respectively. Among the symptoms reported, cough, fever, sore throat and breathlessness were commonest, in 59%, 42%, 20% and 17% SARS-CoV-2 symptomatic patients, respectively (figure 5).

**Table 3.** Distribution of SARS-CoV-2 positive cases (*n* = 9515).

characteristics	Period 1 (P1) 06/04/20 to 17/05/20 (*n* = 1420)	Period 2 (P2) 18/05/20 to 03/09/20 (*n* = 5914)	Period 3 (P3) 04/09/20 to 31/12/20 (*n* = 2181)	total (*n* = 9515)
age (years)				
≤5	24 (1.7)	106 (1.8)	23 (1.0)	153 (1.6)
6–19	208 (14.6)	639 (10.8)	230 (10.6)	1077 (11.3)
20–59	1079 (76.0)	4664 (78.9)	1709 (78.4)	7452 (78.3)
≥60	109 (7.7)	505 (8.5)	219 (10.0)	833 (8.8)
gender				
female	416 (29.3)	1941 (32.8)	726 (33.3)	3083 (32.4)
male	1004 (70.7)	3973 (67.2)	1455 (66.7)	6432 (67.6)
international travel history				
no	1368 (96.3)	5912 (99.97)	2181 (100)	9461 (99.4)
yes	52 (3.7)	2 (0.03)	0	54 (0.6)
institutional quarantine				
no	1113 (78.4)	4656 (78.7)	2008 (92.1)	7777 (81.7)
yes	307 (21.6)	1258 (21.3)	173 (7.9)	1738 (18.3)
state				
Uttar Pradesh	432 (30.4)	5869 (99.2)	1212 (55.6)	7513 (79.0)
Delhi	570 (40.1)	39 (0.7)	969 (44.4)	1578 (16.5)
Madhya Pradesh	418 (29.5)	0	0	418 (4.4)
Ladakh	0	6 (0.1)	0	6 (0.1)
co-morbidity				
no	1396 (98.3)	5908 (99.9)	2181 (100)	9485 (99.7)
yes	24 (1.7)	6 (0.1)	0	30 (0.3)
clinical symptoms				
no	1233 (86.8)	5688 (96.2)	1981 (90.8)	8902 (93.6)
yes	187 (13.2)	226 (3.8)	200 (9.2)	613 (6.4)
hospitalization				
no	1393 (98.1)	5747 (97.2)	2004 (91.9)	9144 (96.1)
yes	27 (1.9)	167 (2.8)	177 (8.1)	371 (3.9)

### 3.3. Associated factors for test positivity for SARS-CoV-2

The raw data are shown in table 4. Age was found to be associated with test positivity. Participants aged 20–59 years and ≥60 years had 46% (adjusted odds ratio (AOR) 1.46 (95% confidence interval (CI): 1.24, 1.73), *p* < 0.001) and 91% higher odds (AOR 1.91 (95% CI: 1.60, 2.28), *p* < 0.001) than participants aged less than 5 years, respectively. The pattern of these age groups that emerged during P2 and P3 wherein only elderlies were found to have a significant association with test positivity (table 5). Sex was found to be associated with SARS-CoV-2 positivity. Overall, the odds of the positive test were more for men as compared to women (AOR 1.08 (95% CI: 1.03, 1.13), *p* < 0.001). There were differences noted among associations for sex in three time frames studied in our study. In P1, higher odds were found for women, and in P3, higher odds were found for men (table 5). Samples from Delhi (AOR 1.19, 95% CI: 1.13,1.27; *p* < 0.001) and Madhya Pradesh (AOR 1.55, 95% CI: 1.38, 1.73; *p* < 0.001) had significantly higher odds of test positivity, compared to samples from Uttar Pradesh (UP). Contrastingly, the odds for samples tested from Ladakh were lesser compared to UP (AOR 0.28, 95% CI: 0.12, 0.63, *p* = 0.002). Recent history of international travel had significantly higher odds (AOR 1.70, 95% CI: 1.26, 2.28; *p* < 0.001). Symptomatic individuals among the entire cohort had significantly higher odds for showing positivity compared to asymptomatic individuals (AOR 1.83, 95% CI: 1.66, 2.02; *p* < 0.001). The presence of co-morbidity and quarantine status were not found to be associated.

**Table 4.** Univariable and multivariable logistic regression analysis determining the association of variables with test positivity for SARS-CoV-2 (*n* = 9515).

explanatory variable	SARS-CoV-2 positivity *n*/*n* (%)	unadjusted		adjusted	
		OR (95% CI)	*p*-value	OR (95% CI)	*p*-value
age (in years)					
≤5	153/2848 (5.4)	1.00		1.0	
6–19	1077/17 379 (6.2)	1.16 (0.98–1.38)	0.088	1.17 (0.99–1.40)	0.068
20–59	7452/96 947 (7.7)	1.47 (1.24–1.73)	<0.001	1.46 (1.24–1.73)	<0.001
≥60	833/8426 (9.9)	1.93 (1.62–2.30)	<0.001	1.91 (1.60–2.28)	<0.001
sex					
female	3083/42 779 (7.2)	1.00		1.0	
male	6432/82 815 (7.7)	1.08 (1.04–1.13)	<0.001	1.08 (1.03–1.13)	<0.001
international travel history					
no	9461/125 207 (7.6)	1.00		1.0	
yes	54/393 (13.7)	1.95 (1.46–2.60)	<0.001	1.75 (1.31–2.34)	<0.001
institutional quarantine					
no	7777/102 624 (7.6)	1.00		1.0	
yes	1738/22 976 (7.6)	1.00 (0.94–1.05)	0.943	1.03 (0.98–1.09)	0.276
state					
Uttar Pradesh	7513/104 744 (7.2)	1.0		1.0	
Delhi	1578/17 345 (9.1)	1.29 (1.22–1.37)	<0.001	1.19 (1.13–1.27)	<0.001
Madhya Pradesh	418/3221 (13.0)	1.93 (1.74–2.14)	<0.001	1.55 (1.38–1.73)	<0.001
Ladakh	6/290 (2.1)	0.27 (0.12–0.61)	0.002	0.28 (0.12–0.63)	0.002
co-morbidity					
no	9485/125 276 (7.6)	1.0		1.0	
yes	30/324 (9.3)	1.24 (0.85–1.81)	0.252	0.71 (0.48–1.04)	0.082
clinical symptoms					
no	8902/121 420 (7.3)	1.0		1.0	
yes	613/4180 (14.4)	2.17 (1.99–2.37)	<0.001	1.83 (1.66–2.02)	<0.001

**Table 5.** Age and sex characteristics of SARS-CoV-2 positive cases among tested over three testing periods.

	Period 1 (P1) 06/04/20 to 17/05/20		Period 2 (P2) 18/05/20 to 03/09/20		Period 3 (P3) ≥04/09/20 to 31/12/20	
	*n*/*n* (%)	OR (95% CI); *p*-value	*n*/*n* (%)	OR (95% CI); *p*-value	*n*/*n* (%)	OR (95% CI); *p*-value
age						
≤5 years	24/359 (6.7)	1.0	106/1315 (8.1)	1.0	23/1174 (2.0)	1.0
6–19 years	208/1995 (10.4)	1.62 (1.05–2.51); 0.030	639/7501 (8.5)	1.06 (0.85–1.32); 0.582	230/7883 (2.9)	1.50 (0.97–2.31); 0.065
20–59 years	1079 (7.8)	1.17 (0.77–1.78); 0.444	4664/50 580 (9.2)	1.15 (0.94–1.41); 0.151	1709/32 494 (5.3)	2.78 (1.83–4.21); <0.001
≥60 years	109/1187 (9.2)	1.41 (0.89–2.23); 0.141	505/4028 (12.5)	1.63 (1.31–2.03); <0.001	219/3211 (6.8)	3.66 (2.37–5.66); <0.001
gender						
female	416/4440 (9.4)	1.0	1941/20 378 (9.5)	1.0	726/17 961 (4.0)	1.0
male	1004/12 974 (7.7)	0.81 (0.72–0.91); 0.001	3973/43 046 (9.2)	0.96 (0.91–1.02); 0.232	1455/26 795 (5.4)	1.36 (1.24–1.50); <0.001

### 3.4. Prospective follow-up of COVID-19 cases

Of the 2158 COVID-19 cases, we could successfully conduct telephone interviews for their health outcomes in 1729 (80%) cases. The mean age (s.d.) of the participants was 33.5 (±15.1) years, and there were 1194 men (69%) in this subset. Of these, 160 (9.2%) participants had co-morbidities. The most common conditions were hypertension and diabetes (39% and 37%, respectively). Current smoking and alcohol intake were reported by 4% and 6% of the participants, respectively. We enquired about the perceived status of weight and height stature as surrogate measures of obesity in this subset of data. Self-reported weight status as perceived by participants was normal for 1365 (79%), lean for 258 (15%) and overweight/obese for 106 (6%). Height was self-perceived as normal for 1352 (78%), short-statured for 120 (7%) and tall statured for 257 (15%). History of more than one family member who had tested as SARS-CoV-2 positive was elicited in 468 (27%) participants.

### 3.5. Clinical symptoms and care

Of 1729 participants, 1272 (74%) remained completely asymptomatic. Of 457 symptomatic, 427 (93.3%) had recovered and were symptomless at the time of telephone interviews. However, 27 (6%) had current symptoms. The two most common symptoms were fever and cough, which were seen in 322 (70.5%) and 236 (52%) participants, respectively. Influenza-like illness (ILI with fever and cough) was reported by 170 (9.8%) participants and complaints of breathlessness were recorded in 60 (13%) patients. Loss of taste or smell was reported by only 13 (3%) persons. All people interviewed recalled their symptoms and status well, perhaps due to the high awareness in the general population of the COVID-19 pandemic. Associated factors with clinical symptom positivity are shown in table 6. Age was found to be significantly associated with symptoms positivity and compared with 0–19 years age group higher odds were found for ages 20–39 years (AOR 1.74, 95% CI: 1.19,2.54, *p* = 0.004), 50–59 years (AOR 2.66, 95% CI: 1.64–4.30, *p* < 0.001) and ≥60 years (AOR 2.20, 95% CI: 1.26,3.86, *p* < 0.005). Patients who reported co-morbidity had two times higher odds for symptom positivity (AOR 2.03, 95% CI: 1.42–2.92, *p* < 0.001). Current smokers exhibited 2.5 times higher odds (AOR: 2.54, 95% CI: 1.37–4.71; *p* = 0.003) compared to non-smokers. Similarly, cases that reported alcohol consumption had almost four times higher odds than non-alcoholics (AOR 3.72, 95% CI: 2.17–6.37, *p* < 0.001). Patients who reported themselves as overweight compared to normal weight cases (AOR 1.83, 95% CI: 1.11–3.00, *p* = 0.016) and short stature compared to patients with normal stature (AOR 1.70, 95% CI: 1.04–2.76) had significantly higher odds ratio indicative of high body mass index associated with symptoms. Participants who reported more family members with SARS-CoV-2 positivity had three times higher odds than those with no such history (AOR 3.08; 95% CI: 2.39, 3.96, *p* < 0.001). Hospitalization was reported by 658 (38%) cases with mean age (s.d.) of 34.4 (±15.8) years of which 447 (68%) were men. In our cohort, only 24 (4%) required oxygenation, 7 (1%) required ventilation while ICU admission was noted for 18 patients (3%).

**Table 6.** Univariable and multivariable logistic regression analysis for associated factors of clinical symptoms with positivity among SARS-CoV-2 positive cases (1729).

explanatory variable	clinical symptoms positivity *n*/*n* (%)	unadjusted odds ratio (95% CI)	*p-*value	adjusted odds ratio (95% CI)	*p*-value
age (years)					
0–19	43/259 (16.6)	1.00		1.00	
20–39	253/936 (27.0)	1.86 (1.30–2.66)	0.001	1.74 (1.19–2.54)	0.004
40–49	64/249 (25.7)	1.73 (1.12–2.68)	0.012	1.52 (0.95–2.42)	0.077
50–59	64/178 (35.9)	2.82 (1.80–4.41)	<0.001	2.66 (1.64–4.30)	<0.001
≥60	33/107 (30.8)	2.24 (1.32–3.78)	0.003	2.20 (1.26–3.86)	0.005
sex					
female	133/535 (24.9)	1.00		1.00	
male	324/1194 (7.1)	1.12 (0.89–1.42)	0.321	1.21 (0.93–1.57)	0.154
co-morbidity					
no	382/1569 (24.4)	1.00		1.00	
yes	75/160 (46.8)	2.74 (1.96–3.81)	<0.001	2.03 (1.42–2.92)	<0.001
smoking					
no	407/1654 (24.6)	1.00		1.00	
yes	50/75 (66.7)	6.12 (3.74–10.03)	<0.001	2.54 (1.37–4.71)	0.003
alcohol intake					
no	390/1628 (23.9)	1.00		1.00	
yes	67/101 (66.3)	6.25 (4.07–9.59)	<0.001	3.72 (2.17–6.37)	<0.001
weight status					
normal	339/1365 (24.8)	1.00		1.00	
lean	61/258 (23.6)	0.93 (0.68–1.28)		0.76 (0.50–1.16)	0.212
overweight	57/106 (53.7)	3.52 (2.35–5.25)	<0.001	1.83 (1.11–3.00)	0.016
height status					
normal	332/1352 (24.5)	1.00		1.00	
short stature	53/120 (44.2)	2.43 (1.66–3.55)	<0.001	1.70 (1.04–2.76)	0.032
tall stature	72/257 (28.0)	1.19 (0.88–1.61)	0.241	1.32 (0.88–1.97)	0.174
intra-familial positivity					
no	257/1261 (20.3)	1.00		1.00	
yes	200/468 (42.7)	2.91 (2.31, 3.66)	<0.001	3.08 (2.39–3.96)	<0.001

## 4. Discussion

This study presents characteristics of a large cohort of 130 132 individuals who were tested for SARS-CoV-2 infection, of whom 125 600 were selected for further analysis. The overall test positivity for SARS-CoV-2 infection was 7.6% while during the study period, it ranged from 4.6% to 9.0% in India. The test positivity rate at a population level is based on the number of tests performed and the stage of transmission within a pandemic setting. In this study, the test samples were received predominantly from Uttar Pradesh, Delhi and Madhya Pradesh. Among the study states, India's capital (Delhi) has recorded maximum positivity from 6.3% to 20.0% during the study period [14]. The test positivity rate in Uttar Pradesh ranged from 2.5% to 6.8% and for Madhya Pradesh it ranged from 3.5% to 9.7% [14] due to changes in disease progression and penetration within interiors of India at different time points. COVID-19 probably spread in cities and urban areas first due to overcrowding and favourable circumstances for spread [15]. There have been three nationwide rounds of sero-surveillance that have reported increasing sero-prevalence in the general population from 0.7% (first round, May–June 2020), 7% (second round, August–September 2020) and 24% (third round, December 2020–January 2021) [15–17]. Our test positivity was maximum (9%) in P2 (May to September 2020) and minimum (5%) during P3 (September onwards). The presented findings reflect the clinic-epidemiological profiles of a heterogeneous population who were suspected to be SARS-CoV-2 infected at varying intensities of infection in respective geographic locations from April to December 2020. Examining through time periods of varying testing guidelines, we observed an increase in younger people, women and more asymptomatic participants being examined for SARS-CoV-2 out of total being testing at NIB during P3. The third nationwide survey also reported an increase in younger people sero-positivity (25%) among 10–17-year-olds surveyed [17]. Overall, test positivity declined over P3 compared to P1 and P2, again resembling nationwide reporting, where test positivity was recorded maximum from June to September and had been declining from October onwards [14].

Participants of all ages were found to be test positive with SARS-CoV-2 and age was found to be positively associated with test positivity and also with exhibiting symptoms. Compared to children, maximum odds for infection positivity were seen in elderly subjects, followed by adults in the age group of 20–59 years. The risk could be explained partially due to concomitant co-morbidities and possible exposure to other infected people within households, who could be asymptomatically infected. This pattern of higher infection rates is consistent with what is reported for the country. Older age groups are considered to have a higher risk of infection and also of severe disease outcomes (like admissions to intensive care units and deaths), as consistently seen in the global and local literature [18,19]. In India, 80% of deaths among SARS-CoV-2 has been reported above 50 years of age group [20]. In our group of participants who were tested, younger age groups were also infected, albeit with lower positivity risks, indicating all age groups are susceptible to infection. In P3, a higher proportion was represented by younger age groups including children and adolescents relative to prior testing time frames probably due to combined efforts of expansive surveillance strategy and widening of opportunities for testing to all persons.

In absolute numbers, more men in our sample were tested and were found to be positive overall, and the odds of test positivity was 6% higher in men compared to women. Male sex has consistently been reported to be an independent risk factor for test positivity [21]. A nationwide sero-surveillance study done in India reported more sero-positivity in men compared to women in the first round, and nearly equal sero-positivity in men (6.7%) and women (6.5%) in the second round [15]. We also found varying risks in different time periods, with almost equal risk in our P2 from May to September 2020. Men have been reported to have severe SARS-CoV-2 infections and higher fatalities than women in India and internationally [5,22]. Varying immunogenic responses have been linked to differentials in response to SARS-CoV-2 infections by sex [23]. Gender differentials also have been reported within local and regional studies within India. We also postulate that there exists a differential in testing utilization rates for men and women, as occurs in Indian communities for a variety of health conditions—there are known variations in patterns of health system utilization by both men and women [24,25]. The sero-survey conducted in Delhi reported a higher sero-positivity in women [26]. Our sample of participants included 42 779 women but overall comprised only approximately one-third of the entire sample tested. Reasons for differentials in testing between men and women remain an important area for in-depth qualitative enquiry. It has been reported in two southern Indian states (Tamil Nadu and Andhra Pradesh) through contact tracing and active finding that contacts were younger and more often women than index cases. Also, the same study reported a secondary attack rate of 11% for high-risk close contacts [27].

Participants with a history of international travel had significantly higher odds of test positivity in our sample. India in its active response institutionalized a strong and early tracking system by issuing travel advisories, screening and testing of passengers, and curbing international travel at a later stage for preventing transmission of the disease. As the indigenous transmission expanded, fewer cases with international travel history were reported. The guidelines and indications for testing have been periodically revised over the course of the pandemic in India [9,10,13]. Travel-related spread has once again gained importance against the backdrop of increasing transmission in European countries and newer variants or mutational strains accelerating the local spread. The Government of India has issued recent advisories curbing international travel from select nations where enhanced transmission of SARS-CoV-2 has been reported [28]. Also, there is now provision for genomic surveillance and additional tracking of mutant strains of SARS-CoV-2 [28–31]. Further, web-based COVID tracking dashboards like PRACRITI make predictions of the subsequent three weeks in terms of active cases along with basic reproduction number (R0) values for each state in India [32]. The PRACRITI predictions for the second wave of infections that are ongoing (April 2021 onwards) are very alarming.

Significantly, participants with clinical symptoms had two times higher odds of test positivity. Cough and fever were the two most common symptoms reported in our symptomatic patients followed by sore throat and breathlessness. Similar symptoms have also been reported by other international and Indian studies [5,6,22,33–36]. There have been differences in the profile of participants in settings outside India where the mean age of participants is on the older side, as compared to Indian settings, where younger age groups are affected more due to pre-existing population demographic profile. In our study, the mean age of participants was 33 years. It is noteworthy that for every symptomatic positive case for SARS-CoV-2, there were approximately six times more symptomatic persons with reasons other than SARS-CoV-2. Also, there was a large proportion of asymptomatic infective positive cases in our group of participants. For every one positive case with symptoms, approximately 16 cases were symptomless. A high proportion of asymptomatic SARS-CoV-2 positive cases have also been reported in hospitalized COVID-19 and other reports from India [34–36]. It is already established that there is a high percentage of asymptomatic cases with COVID-19, and these, when undetected, pose significant challenges for the containment of the virus [37–39]. Epidemiological analysis in the state of Karnataka reported predominantly asymptomatic cases in the younger age group of 16–45 years and symptomatic cases in the age band of 31–65 years. The study also suggested that both asymptomatic and symptomatic cases contribute to the spread of infection [40].

Sero-surveillance studies within the country suggest high infection load in select regions of the country though actual cases confirmed via testing are strikingly lower in comparison. It has been reported that for every case there are between 27 and 31 infections in the community [15,17,27]. Considering an adjustment factor of 27 on a conservative side, the actual infected cases up to 31 December 2020 in our study areas would be 16.8 million in Delhi (against 0.6 million cases reported), 15.8 million in Uttar Pradesh (against 0.5 million cases reported) and 6.5 million in Madhya Pradesh (against 0.2 million cases reported) respectively. Thus, the potential to go undetected (and possibly still spread the virus) is very high in the Indian context. Another peculiar pattern, though on a positive note, that has been reported in India is the overall high recovery rate and lower fatalities [41]. Nonetheless, co-morbidities have been found to be associated with the expression of symptoms in our sample. Cases with co-morbidities have been found to be associated with severe illnesses requiring hospitalization and critical care (and include fatalities) [22,42,43]. In any case, the claims of a low fatality rate in India are unreliable due to incomprehensive testing and lack of availability of data on death records for analysis.

In this work, lifestyle habits like smoking and alcohol consumption appeared as independent risk factors in symptomatic patients. Smoking is already linked to severe COVID-19 illness and fatalities [44]. Both smoking and alcohol can mediate a heightened inflammatory response and weaken host immune defences [45]. Indeed, the extended periods of lockdown and then release could have promoted alcohol abuse [46]. We also found excess weight as an independent factor with higher odds for developing COVID-19 symptoms. We were unable to measure body mass index, and hence, this association may be interpreted with caution. Nevertheless, obesity has been linked with poor immunity and leads to poorer outcomes [47]. Strikingly, our data suggest higher familial SARS-CoV-2 case positivity is significantly associated with increased odds for developing symptoms. Familial clustering does increase disease transmission, including from asymptomatic individuals who aid disease spread [48]. This finding reiterates the pivotal role of contact tracing and subsequent isolation/quarantine of family members in halting transmission of the virus. In the Indian context, this insight is critical given that most households are typified by individuals who share living spaces and facilities. During self-isolation, pulse oximetry also became an integral component of home-based COVID-19 patients' respiratory disease management [49].

Our study has several strengths. It is based on a very large sample of 125 600 participants who got tested for SARS-CoV-2 and it included all age groups. However, this cohort represents only those who had access to healthcare services and presented themselves for testing. We lack data on several parameters that influence health and disease outcomes including socio-economic index, urban/rural status, poverty and vulnerability status. We do not have information pertaining to the exact source of exposure in our cases. We were constrained by the non-availability of data through record reviews as these data are collected at a high-volume testing centre, keeping in mind the resource and management constraints. Future studies should include outcome data, especially in the context of India, where there are wide social and economic disparities. We followed up a small fraction of positive cases in the initial part of the data collection period and that provided valuable insights on associated factors with symptomatic cases. Our patients largely had a milder spectrum of disease, a pattern that again is more generalizable in the Indian context due to unknown reasons except for overall young demography. Our findings are based on self-reports and data information filled at the time of requisition of tests. We have excluded information from participants whose information was deficient. Also, predominantly asymptomatic infections are obtained in our sample that was being tested. It was difficult for us to truly differentiate between pre-symptomatic and symptomatic infections; as had been reported earlier in the literature, people who had been tested may have been asymptomatic at the time of obtaining the sample but may exhibit symptoms in their future clinical course of infection [36,50]. This will require conducting prospective studies on these asymptomatic patients and will inform our understanding of symptom and outcome status better in these infected individuals.

In sum, we found a 7.6% positivity rate in a large cohort of those tested for SARS-CoV-2 between April and December 2020 in India. Most of the positive patients were asymptomatic, with cough and sore throat being the commonest symptoms reported. In our follow-up sub-study, a large majority of COVID-19 cases recovered fully with only 2% continuing with symptoms. Concomitant disease, smoking, drinking, obesity and familial COVID-19 patients were independent risk factors for expressing symptoms among diseased samples. Our findings have several implications for programmatic action directed at COVID-19 containment. As a majority of COVID-19 patients are asymptomatic as per this cohort, it is thus imperative to intensify efforts towards testing, identification and isolation of cases. It is noteworthy that all ages were found susceptible to infection, including children and adolescents (less than 19 years). Thus, public health workforces should execute household-level surveillance for case detection targeting all age groups. Significant factors associated with test positivity were increasing age, male sex, international travel and having symptoms. Co-morbidity was significantly associated with the exhibition of symptoms. There is still a considerable area and population in India that is susceptible to SARS-CoV-2 infection and risk factors obtained above must be given due attention for testing and surveillance operations. India, with a considerable number of young people, who have not been infected to a large extent, remains vulnerable, especially in the context where mutant variants have been reported from other parts of the world that have demonstrated higher transmissibility and propensity to infect young people [51]. The clinic-epidemiological profiles presented here will be more valuable with comparative data from other parts of India and from other COVID-19-afflicted regions of the world. Also, the COVID-19 mitigation steps taken by the government have provided a blueprint for other infectious diseases [52,53]. As is evident through past sero-surveys, a high proportion of the population in India, including in the rural areas, remains vulnerable to acquiring SARS-CoV-2 infection, as is evident from the second wave of infections. The subsequent waves may infect even larger remaining populations (driven by current and new variants that will arise) and thus only widespread and rapid immunization campaigns can protect the masses in India. The planning and roll-out of COVID-19 vaccines in India for its masses are hampered and severely delayed due to pandemic mismanagement that has also resulted in severe shortages of oxygen, hospital beds, ICUs, medicines and healthcare workers. Vaccination is thus proving to be a huge challenge for the already frail Indian healthcare system, and India may consider valuable lessons from success stories like the polio vaccination drive [54]. Studies like our current work will be imperative for inferring trends of disease spread in subsequent waves of infections, especially in the backdrop of concomitant immunizations and the evolution of new variants of concern.

## Data accessibility

This paper presents new data.
